# Investigation of protein secretion and secretion stress in *Ashbya gossypii*

**DOI:** 10.1186/1471-2164-15-1137

**Published:** 2014-12-18

**Authors:** Tatiana Q Aguiar, Orquídea Ribeiro, Mikko Arvas, Marilyn G Wiebe, Merja Penttilä, Lucília Domingues

**Affiliations:** CEB – Centre of Biological Engineering, University of Minho, 4710-057 Braga, Portugal; Department of Life Sciences, Imperial College London, Exhibition Road, London, SW7 2AZ UK; VTT Technical Research Centre of Finland, Espoo, P.O. Box 1000, FIN-02044 VTT Finland

**Keywords:** *Ashbya gossypii*, Proteins secretion, Secretion stress, Secretome, Transcriptome

## Abstract

**Background:**

*Ashbya gossypii* is a filamentous Saccharomycete used for the industrial production of riboflavin that has been recently explored as a host system for recombinant protein production. To gain insight into the protein secretory pathway of this biotechnologically relevant fungus, we undertook genome-wide analyses to explore its secretome and its transcriptional responses to protein secretion stress.

**Results:**

A computational pipeline was used to predict the inventory of proteins putatively secreted by *A. gossypii* via the general secretory pathway. The proteins actually secreted by this fungus into the supernatants of submerged cultures in minimal and rich medium were mapped by two-dimensional gel electrophoresis, revealing that most of the *A. gossypii* secreted proteins have an isoelectric point between 4 and 6, and a molecular mass above 25 kDa. These analyses together indicated that 1-4% of *A. gossypii* proteins are likely to be secreted, of which less than 33% are putative hydrolases. Furthermore, transcriptomic analyses carried out in *A. gossypii* cells under recombinant protein secretion conditions and dithiothreitol-induced secretion stress unexpectedly revealed that a conventional unfolded protein response (UPR) was not activated in any of the conditions, as the expression levels of several well-known UPR target genes (e.g. *IRE1*, *KAR2*, *HAC1* and *PDI1* homologs) remained unaffected. However, several other genes involved in protein unfolding, endoplasmatic reticulum-associated degradation, proteolysis, vesicle trafficking, vacuolar protein sorting, secretion and mRNA degradation were up-regulated by dithiothreitol-induced secretion stress. Conversely, the transcription of several genes encoding secretory proteins, such as components of the glycosylation pathway, was severely repressed by dithiothreitol

**Conclusions:**

This study provides the first insights into the secretion stress response of *A. gossypii*, as well as a basic understanding of its protein secretion potential, which is more similar to that of yeast than to that of other filamentous fungi. Contrary to what has been widely described for yeast and fungi, a conventional UPR was not observed in *A. gossypii*, but alternative protein quality control mechanisms enabled it to cope with secretion stress. These data will help provide strategies for improving heterologous protein secretion in *A. gossypii*.

**Electronic supplementary material:**

The online version of this article (doi:10.1186/1471-2164-15-1137) contains supplementary material, which is available to authorized users.

## Background

The protein secretory pathway is an important area of fungal research, as the secretion of proteins by fungal cells is of major biological and commercial significance. *Ashbya gossypii* (syn. *Eremothecium gossypii*), a well known industrial producer of riboflavin [1], is a filamentous fungus that has been recently considered as a host for the production of recombinant proteins [2]. However, its protein secretory pathway and the spectrum of proteins natively secreted by this fungus to the extracellular space remain virtually unexplored. *A. gossypii* has one of the smallest eukaryotic genomes known [3] and is phylogenetically closer to yeast than to other filamentous fungi [4], sharing a high degree of gene homology and gene order conservation with the budding yeast *Saccharomyces cerevisiae* [3]. Although efficient protein secretion is generally associated with filamentous growth, the secretion levels of the heterologous proteins endoglucanase I (EGI) and cellobiohydrolase I (CBHI) from *Trichoderma reesei* in *A. gossypii* were previously reported to be low [2]. The production of heterologous proteins by fungal species is usually much less efficient than the production of native proteins and several steps in the secretory pathway (e.g. translation, translocation, folding, transport and secretion) are potential bottlenecks for heterologous protein production [5–8].

In eukaryotes, newly synthesised proteins are typically targeted for entry into the general secretory pathway by the presence of a N-terminal signal sequence that typically has a length between 15 to 30 amino acids and comprises a central hydrophobic region flanked by hydrophilic N- and C- terminal regions [9]. Taking advantage of the characteristics of these signal peptides and other sorting signals, several computational tools have been developed to predict the subcellular location of proteins such as the extracellular space [10–12]. These have been used for the genome-wide prediction of putative fungal secretomes [13]. As the translocation of proteins into the endoplasmatic reticulum (ER) is determined by the secretion signal, the correct processing of signal peptides, together with the proper folding of proteins within the ER, is important in recombinant protein production and secretion [14, 15].

The ER serves as the first station of the secretory pathway. Its lumen provides a unique oxidizing environment in which highly active folding machinery, including molecular chaperones and foldases, facilitates and promotes the folding, assembling, modification and maturation of proteins. To ensure that only properly folded proteins move onward through the secretory pathway, the ER also contains stringent quality control mechanisms that retain malfolded (unfolded or misfolded) proteins and ultimately retrotranslocate them into the cytosol for proteasomal degradation through a process called ER-associated degradation (ERAD) (reviewed in [16]). Environmental and physiological demands (e.g. cell differentiation, pH and temperature, nutrient limitation, expression of heterologous proteins, etc.) can lead to an imbalance between the protein folding load and the protein folding capacity in the ER lumen, resulting in an accumulation of malfolded proteins, i.e. ER stress [17–19]. In response to ER stress, eukaryotic cells have evolved signalling pathways that induce the unfolded protein response (UPR). The UPR activates a gene expression program that helps to restore homeostasis in the ER by enhancing ER protein folding capacity and ERAD, and reducing translation and entry of new proteins into the ER (reviewed in [20, 21]).

Cellular responses to the accumulation of malfolded proteins in the ER have been described for yeast, filamentous fungi and higher eukaryotes, and shown to play a significant role in the stress response to production of secreted recombinant proteins [14, 22]. The inositol-requiring enzyme 1 (*IRE1*) gene encodes the protein that controls the most conserved and best understood UPR signalling pathway in lower eukaryotes [23]. Ire1p has a luminal sensing domain coupled to cytosolic kinase and endoribonuclease (RNase) domains [24]. The accumulation of malfolded proteins in the ER lumen leads to the oligomerization of the Ire1p luminal domain and thereby to the activation of its kinase and RNase functions [21].

Upon Ire1p activation, the Ire1p RNase initiates the splicing of a non-conventional intron from *HAC1* messenger RNA (mRNA), thus allowing the translation of active Hac1p, a basic-leucine zipper (bZIP) transcription factor that specifically binds to UPR elements (UPREs) in the promoter region of UPR target genes, thereby up-regulating their transcription [25, 26]. In response to strong ER stress, Ire1p signaling may also cause regulated *IRE1*-dependent mRNA decay (RIDD) to reduce the ER load by inducing the degradation of mRNAs encoding secretory proteins [21, 27, 28]. Several UPR target genes have been identified, among which are those encoding ER chaperones and protein folding enzymes, ER structural and transport proteins, members of the ERAD machinery and components that mediate autophagy [29–31]. The bZIP transcription factor Gcn4p, a major controller of the amino acid starvation response, has been shown to also play an essential role in the induction of a large subset of these target genes during ER stress, by directly interacting with Hac1p and modulating its activity in an *IRE1*-independent way [32, 33].

The characterization of protein secretory pathway components and of the regulatory range of secretion stress responses in yeast and filamentous fungi has often relied on inducing ER stress with chemical secretion blockers such as the folding inhibitor dithiothreitol (DTT), the glycosylation inhibitor tunicamycin and the protein trafficking inhibitor brefeldin A. In the present study, we analysed the events taking place at the transcription level in *A. gossypii* under recombinant protein secretion conditions and also under DTT-induced secretion stress, in order to identify bottlenecks that may hamper protein secretion in *A. gossypii*. Moreover, to explore the native proteins putatively secreted by this fungus, we also analysed its predicted secretome by combining comparative *in silico* predictions for classically secreted proteins with experimental data derived from two-dimensional (2-D) gel electrophoresis.

## Results

### The *A. gossypii*secretome

The *A. gossypii* secretome was predicted from an analysis of its genomic data, using a computational pipeline (see Materials and Methods) to detect known cellular sorting and localization signals in its putative proteins. Of the 4,776 open reading frames (ORFs) annotated in the *A. gossypii* genome, 333 (7%) were predicted to encode proteins containing a N-terminal signal peptide (Additional file 1: Table A1.1), and thus to enter the general secretory pathway. However, only 54 proteins (1% of the *A. gossypii* total proteome) were predicted to be secreted extracellularly by the computational pipeline used (Additional file 1: Table A1.2), the others being targeted to different cellular compartments (Additional file 1: Table A1.1). All of the 54 proteins in the *A. gossypii* predicted secretome have homologs in closely related Saccharomycotina species, but 7 have no homologs in *S. cerevisiae*. 67% were predicted to contain at least one *N*-glycosylation site (Additional file 1: Table A1.2). Enzymes predicted to have hydrolytic activity comprised 33% of the *A. gossypii* predicted secretome (Additional file 1: Table A1.2).

### Growth and protein secretion by *A. gossypii*

*A. gossypii* ATCC 10895 produced 5.7 ± 0.2 g/l dry biomass in defined minimal medium (DMM) and 8.1 ± 0.3 g/l in rich medium (AFM) with sucrose as primary carbon source. At the beginning of the stationary phase the supernatant of the culture growing in minimal medium contained a total protein content of 130 mg/l and that of the culture growing in complex medium contained 218 mg/l.

**Figure 1:**
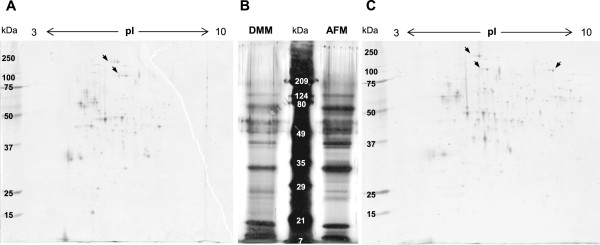
**Electrophoretic profiles of the proteins secreted by** ***A. gossypii*** **into the culture supernatant.** Panels **A** and **C** show representative 2-D electrophoresis gels of *A. gossypii* culture supernatants derived from bioreactor batch cultures in minimal (DMM) and rich (AFM) medium, respectively. Pannel **B** shows the SDS-PAGE gel of the same supernatants. The arrows indicate spots that are most likely diffent isoforms of the same protein.

Sodium dodecyl sulfate polyacrylamide gel electrophoresis (SDS-PAGE) showed 12 distinct protein bands in the culture supernatant of both DMM and AFM cultures, ranging from 7 kDa to 209 kDa (Figure 1B). On 2-D gels, 18 protein spots were visible at higher abundance in the culture supernatants and were common to both DMM and AFM cultures (from a total of 101 spots common to both). Of these, at least 2 were obviously isoforms of other protein spots with identical molecular weight but different isoelectric points (Figures 1A and C). As shown in Figures 1A and C more protein spots were detected in AFM (182) than in DMM (157) culture supernatants. In addition, the distribution of the protein spots on the 2-D gels indicated that most of the *A. gossypii* secreted proteins have isoelectric points between 4 and 6, and molecular weights above 25 kDa, although some proteins present only in AFM cultures did have slightly higher isoelectric points (6–8).

### Effect of DTT on the *A. gossypii*growth

**Figure 2:**
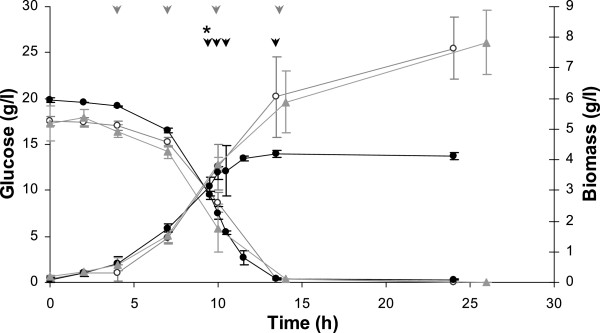
**Glucose consumption (** ***solid lines*** **) and growth (** ***dashed lines*** **) of recombinant** ***A. gossypii*** **in batch cultures in AFM with G418 at pH 6.0, 30°C and 500 rpm, with 1.0 vvm aeration.** (○) EGI producing strain, (▲) empty vector strain and (●) DTT-stressed EGI producing cells. Values represent the average ± standard deviation of two independent bioreactor cultures. Grey arrows indicate the sampling times for non-treated cultures and black arrows the sampling points for DTT-treated cultures. *Indicates the time at which DTT was added to EGI producing cultures.

In order to study the effects of secretion stress on *A. gossypii* growth and gene transcription, duplicate bioreactor batch cultivations were carried out with a recombinant EGI producing strain (previously reported to secrete low levels of this heterologous protein [2]), its corresponding empty vector control strain, and the EGI producing strain treated with a well known secretion stress inducer, DTT (Figure 2). As previously described [2], the production of EGI alone did not alter cellular growth. Conversely, the addition of 10 mM final concentration DTT to cultures after 9.5 h caused a substantial and immediate reduction in the specific growth rate of the *A. gossypii* EGI producing cells (Figure 2). When the same concentration of DTT was added after only 6 h, the *A. gossypii* cells immediately stopped growing (data not shown).

### Effect of recombinant protein secretion on the *A. gossypii*transcriptome

The overall variation in the microarray gene expression profiles between *A. gossypii* cells secreting recombinant EGI and those which did not was very small. LIMMA (i.e. a modified *t*-test) could not detect significant differences between gene expression of the EGI secreting and the empty vector control strains at any time. However, in order to directly study the link between EGI secretion and gene expression, the correlation of each gene’s expression to EGI secretion in EGI producing cultivations was calculated. As the correlation analysis included the variation in replicates of single samples (unlike LIMMA), it was expected to be more sensitive than LIMMA. Twenty-one genes were found to be differentially expressed (FDR of 4.4%) using the correlation approach, of which 16 were up-regulated and 5 down-regulated in the strain secreting recombinant protein (Table 1). Gene ontology (GO) enrichment analyses for this set of genes hinted at translation down-regulation and ion and amino acid transmembrane transport up-regulation having occurred during EGI secretion.

**Table 1:** **Genes differentially regulated in** ***A. gossypii*** **during the production of recombinant EGI (FDR of 4.4%)**

	***A. gossypii***gene	***S. cerevisiae***homolog(s)	Predicted protein function	Biological processes
**Up-regulated**	*AAR030W*	*CTR1*	High-affinity copper transporter	Amino acid transport
	*AAR080W*	No homolog	Unknown	
	*ADL123C*	*PHO4*	Transcription factor that activates transcription cooperatively with Pho2p in response to phosphate limitation	
	*ADL153W*	*RRI2*	Subunit of the COP9 signalosome complex	
	*ADR080W*	*FRE1*	Ferric reductase and cupric reductase	Iron transmembrane transport
	*AEL294C*	*FTR1*	High-affinity iron permease	
	*AER428W*	*OM45*	Major constituent of the mitochondrial outer membrane	
	*AFL135W*	*YMR181C, YPL229W*	Unknown	
	*AFR156W*	*PUT4*	High-affinity proline permease	Transmembrane transport
	*AFR442C*	*PHO84*	High-affinity inorganic phosphate transporter and low-affinity manganese transporter	
	*AFR529W*	*SUC2*	Invertase	
	*AFR595W*	*MCH1*	Protein with similarity to mammalian monocarboxylate permeases	
	*AFR668W*	*CAN1, ALP1*	Plasma membrane arginine permease	Ion transport
	*AFR739C*	No homolog	Unknown	
	*AGL097C*	*ENA2, ENA5, ENA1*	P-type ATPase sodium pump, involved in Na^+^ and Li^+^ efflux to allow salt tolerance	
	*AGR304W*	*MTH1, STD1*	Protein involved in the control of glucose-regulated gene expression	
**Down-regulated**	*ABL065W*	*RPG1*	Subunit of the core complex of translation initiation factor 3 (eIF3)	Regulation of translation
	*ABL174C*	*SSB2, SSB1*	Cytoplasmic ATPase that is a ribosome-associated molecular chaperone; may be involved in the folding of newly-synthesized polypeptide chains; member of the HSP70 family	Posttranscriptional regulation of gene expression
	*AEL032W*	*GCN20*	Positive regulator of the Gcn2 kinase activity	Regulation of cellular protein metabolic process
	*AER366W*	*FLX1*	Protein required for transport of FAD across the mitochondrial membrane	Regulation of translational elongation
	*AGR261W*	*RPS28B, RPS28A*	Protein component of the small ribosomal subunit	

The corresponding *S. cerevisiae* homologs are indicated, as well as the predicted functions. The biological processes enriched (p < 0.01) in the up- and down-regulated gene clusters are also indicated.

Expression of the *eglI* gene itself (included in the microarray although not part of the *A. gossypii* genome) in the EGI producing cells was around 5 fold higher than the background signal of the gene in the non-producing cells, which contained the empty vector, and approximately 14 fold lower than the expression levels of *TEF.*

### Effect of DTT-induced stress on the *A. gossypii*transcriptiome

**Figure 3:**
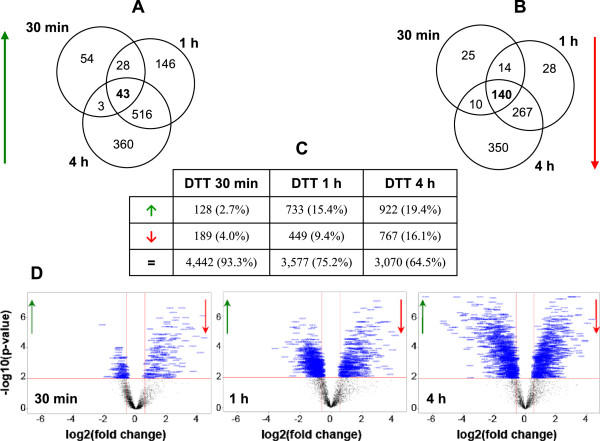
**Overall variations in the** ***A. gossypii*** **transcriptome after addition of DTT.** The data refers to the outcome of the LIMMA analysis for differentially expressed genes within 30 min, 1 h and 4 h of exposure to DTT, when compared to the time immediately before DTT addition (fold change > 1.5 and p-value < 0.01). The Venn diagrams indicate the number of genes that had increased **(A)** or reduced **(B)** expression after 30 min, 1 h and 4 h of exposure to DTT. In table **(C)** the absolute number and relative percentage (between brackets) of genes transcriptionally up-regulated (↑), down-regluated (↓) and unchanged (=) by DTT after different exposure times are indicated. The Volcano plots **(D)** obtained from the LIMMA analysis show the overall significant fold changes in the *A. gossypii* gene expression profiles after 30 min, 1 h and 4 h of DTT addition. Negative fold changes represent genes with increased (↑) expression and positive fold changes genes with reduced (↓) expression after exposure to DTT.

Upon addition of DTT to cultures of *A. gossypii* EGI producing cells during logarithmic growth, the gene expression profile changed significantly (Figure 3), as did the growth rate (Figure 2). When comparing the transcript levels of all genes at the time immediately before the addition of DTT (9.5 h after inoculation and defined as time zero for DTT addition) with those at 30 min, 1 h and 4 h after DTT addition, 128 genes were already up-regulated and 189 down-regulated after 30 min of exposure to DTT (Figure 3C). The up-regulation of 43 of these genes was sustained up to 4 h of treatment (Figure 3A), and the same was observed for 140 of the 189 down-regulated genes (Figure 3B). As can be seen from Figure 3C, DTT induced more genes than it repressed. However, down-regulation was greater than up-regulation after 30 min and 1 h of DTT treatment, as higher fold changes were observed in the transcript levels of down-regulated than up-regulated genes (Figure 3D).

**Figure 4:**
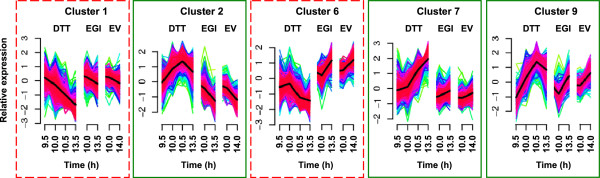
**Expression profiles of the co-expression clusters whose profiles changed differently in the DTT-treated** ***vs*** **. non-treated recombinant cells.** DTT was added at 9.5 h to bioreactor cultures of the EGI producing strain and expression analyses were performed after 30 min (10 h of culture), 1 h (10.5 h of culture) and 4 h (13.5 h of culture) of exposure. The gene expression variation of the non-treated EGI producing strain (EGI) and corresponding empty vector strain (EV) from 10 to 14 h of cultivation are shown for comparison. Dashed red squares indicate gene clusters down-regulated by DTT and green squares represent clusters up-regulated by DTT.

DTT caused wide-ranging effects on *A. gossypii* gene expression profiles (Tables 2, 3 and 4). Some of the changes probably correlate with the observed reduction in growth rate (Figure 2). For instance, within 30 min, the transcription of genes involved in filamentous growth (Table 3), glycosylation and lipoprotein biosynthesis (Cluster 1 in Figure 4, Tables 2 and 3), and cell wall biosynthesis was already down-regulated by DTT. A down-regulation of ribosomal protein-encoding genes has been correlated with reductions in growth rate of *S. cerevisiae* [34, 35] and analysis of the co-expression clusters revealed that genes encoding ribosomal proteins were also down-regulated in DTT treated *A. gossypii* cells compared to the control, particularly after 1 h of exposure to DTT (Cluster 1 in Figure 4, Table 2).

**Table 2:** **Biological processes enriched (p < 0.01) in the co-expression clusters whose expression profiles varied differently in the DTT-treated** ***vs*** **. non-treated recombinant cells**

Cluster 1	Cluster 6
Ion transport	Reproduction
Ribosomal large subunit biogenesis	Response to pheromone
Glycosylation	Ascospore wall assembly
Glycoprotein metabolic process	mRNA splicing, via spliceosome
Macromolecule modification	Conjugation
Cellular iron ion homeostasis	M phase
Attachment of GPI anchor to protein	Peptide transport
Lipoprotein metabolic process	DNA recombination
Translational elongation	External encapsulating structure organization
Sulfur amino acid transport	Regulation of microtubule polymerization or depolymerization
Peptidyl-diphthamide biosynthetic process from peptidyl-histidine	
	DNA metabolic process
	Cell wall assembly
	RNA splicing
**Cluster 2**	**Cluster 7**
Reproduction	Endocytosis
Organelle organization	Response to biotic stimulus
Response to pheromone	Positive regulation of homeostatic process
Growth	Proteasome assembly
Multi-organism process	Purine ribonucleoside catabolic process
Biological regulation	Cell division
Transcription elongation from RNA polymerase II promoter	NAD biosynthesis via nicotinamide riboside salvage pathway
Conjugation	
Gene expression	Amide biosynthetic process
Regulation of transcription during mitosis	Response to singlet oxygen
Mitotic cell cycle	Membrane invagination
Actin filament-based process	Response to osmotic stress
Isoleucyl-tRNA aminoacylation	Response to abiotic stimulus
Regulation of protein catabolic process	
Protein localization to organelle	**Cluster 9**
Chromosome segregation	Developmental process involved in reproduction
Small GTPase mediated signal transduction	Vacuolar transport
Cellular localization	Negative regulation of biological process
Regulation of localization	Response to stimulus
Cell cycle	Autophagy
DNA-dependent transcription elongation	DNA-dependent transcription initiation
Proteolysis	Cellular membrane fusion
Transcription from RNA polymerase II promoter	Vacuolar protein processing
Macromolecule localization	Post-translational protein modification
Cellular macromolecule biosynthetic process	Cellular response to stress
Regulation of biological process	Transcription initiation from RNA polymerase III promoter
Nucleus organization	Macromolecule localization
Regulation of cell size	Cytokinesis
Transmembrane transport	DNA metabolic process
mRNA-binding (hnrnp) protein import into nucleus	Vesicle-mediated transport
Nuclear pore organization	Negative regulation of metabolic process
Nucleosome disassembly	Vacuole organization
	Cell communication
	Meiotic mismatch repair
	Response to extracellular stimulus

Clusters 1 and 6 were down-regulated by DTT, whereas clusters 2, 7 and 9 were up-regulated by DTT (Figure 4).

**Table 3:** **Biological processes enriched (p < 0.001) in the gene clusters significantly down-regulated (fold change > 1.5 and p-value < 0.01) after 30 min, 1 h and 4 h of DTT treatment, in comparison with the time immediately before addition of DTT**

30 min	1 h	4 h
***Thiamine transport***	‘De novo’ IMP biosynthetic process	Adenine salvage
Filamentous growth	***Cadmium ion transport***	***Cadmium ion transport***
***Response to copper ion***	***Response to copper ion***	***Response to copper ion***
Phytochelatin biosynthetic process	Cell-cell adhesion	Organophosphate metabolic process
Lipid metabolic process	Iron assimilation	Polyphosphate metabolic process
Barrier septum assembly	Polyphosphate metabolic process	Cellular oligosaccharide metabolic process
Positive regulation of catabolic process	**N** **-glycan processing**	
	Peptide biosynthetic process	Group II intron splicing
Carbohydrate metabolic process	Glutamine metabolic process	Triglyceride biosynthetic process
Hydrogen peroxide metabolic process	**Acylglycerol biosynthetic process**	Mannoprotein biosynthetic process
	**Organic alcohol transport**	***Thiamine transport***
Glycoprotein metabolic process	***Thiamine transport***	Cellular biosynthetic process
Alditol biosynthetic process	Endonucleolytic cleavage to	Regulation of translational fidelity
	generate mature 3′-end of ssu-rRNA from (ssu-rRNA, 5.8 s rRNA, lsu-rRNA)	
Purine ribonucleoside monophosphate metabolic process		***Vitamin transport***
		Glycoprotein metabolic process
**Acylglycerol biosynthetic process**		***Lipid storage***
**Organic alcohol transport**	***Lipid storage***	Septin checkpoint
Nucleoside transport	**Organic ether metabolic process**	Protein metabolic process
***Lipid storage***	***Vitamin transport***	Box c/d snoRNA metabolic process
***Cadmium ion transport***	***Nucleoside monophosphate metabolic process***	Nucleoside metabolic process
Alcohol metabolic process		***Nucleoside monophosphate metabolic process***
***Vitamin transport***	Ctp metabolic process	
***Nucleoside monophosphate metabolic process***	**Nucleobase-containing compound biosynthetic process**	**Glycosylation**
		Regulation of mating-type specific transcription, DNA-dependent
**Glycosylation**	Glycerol ether metabolic process	
**Organic ether metabolic process**		Cell wall glycoprotein biosynthetic process
Lipoprotein biosynthetic process		
Purine base biosynthetic process		Purine nucleoside metabolic process
Lipoprotein metabolic process		
Deadenylation-dependent decapping of nuclear-transcribed mRNA		
Nucleobase metabolic process		
Glycerol ether metabolic process		
Cellular carbohydrate metabolic process		
***N*** **-glycan processing**		
Coenzyme a biosynthetic process		
**Nucleobase-containing compound biosynthetic process**		

GO terms highlighted in bold were overrepresented at two or all (bold italic) DTT exposure times.

**Table 4:** **Biological processes enriched (p < 0.001) in the gene clusters significantly up-regulated (fold change > 1.5 and p-value < 0.01) after 30 min, 1 h and 4 h of DTT treatment, in comparison with the time immediately before addition of DTT**

30 min	1 h	4 h
Spermine biosynthetic process	***Protein unfolding***	DNA dealkylation involved in DNA repair
Interspecies interaction between organisms	Bipolar cellular bud site selection	
	**Early endosome to golgi transport**	Carbon utilization
S-adenosylmethionine transport	Membrane docking	Asexual reproduction
Traversing start control point of mitotic cell cycle	***Trehalose biosynthetic process***	Response to stimulus
	Phosphorus metabolic process	**Negative regulation of transferase activity**
Macromolecule metabolic process	Regulation of DNA repair	
Glucose 1-phosphate metabolic process	Fructose transport	**Early endosome to golgi transport**
	Asymmetric protein localization	Cellular aldehyde metabolic process
Cis assembly of pre-catalytic spliceosome	Vesicle-mediated transport	NAD biosynthesis via nicotinamide riboside salvage pathway
	mRNA polyadenylation	
**Macromolecule catabolic process**	Cellular localization	***Trehalose biosynthetic process***
***Protein unfolding***	Cell wall macromolecule catabolic process	***Protein unfolding***
Golgi localization		Nuclear mRNA 5′-splice site recognition
Leading strand elongation	**Secretion**	
Cellular macromolecule metabolic process	Macromolecule localization	Asymmetric protein localization
	Cofactor transport	**Secretion**
Negative regulation of transcription from RNA polymerase II promoter during mitosis	**Oligosaccharide metabolic process**	Sodium ion transport
	**Macromolecule catabolic process**	**Negative regulation of developmental process**
	Positive regulation of lipid metabolic process	
**Proteolysis**		**Oligosaccharide metabolic process**
Transcription initiation, DNA-dependent	Transcription from RNA polymerase II promoter	Tetrapyrrole catabolic process
		Intron homing
Stress-activated protein kinase signaling cascade	**Negative regulation of catalytic activity**	**Negative regulation of catalytic activity**
**Negative regulation of transferase activity**	**Regulation of hydrolase activity**	**Regulation of hydrolase activity**
	**Regulation of transferase activity**	**Regulation of transferase activity**
***Trehalose biosynthetic process***	**Proteolysis**	Glycerophospholipid catabolic process
	Regulation of response to stimulus	
	**Negative regulation of developmental process**	

GO terms highlighted in bold were overrepresented at two or all (bold italic) DTT exposure times.

Treatment of *A. gossypii* cells with DTT led to major repression of the protein glycosylation pathway (in particular of the *N*-glycosylation pathway) (Tables 2, 3 and Additional file 2). The observed repression was not only at the ER, but also at the Golgi processing level (Additional file 2), indicating that a major accumulation of unglycosylated proteins may have occurred. Genes involved in response to stress, transcription, protein unfolding, proteasome assembly, proteolysis, vesicle trafficking, vacuolar protein sorting, secretion, trehalose biosynthesis and DNA repair were induced by DTT (Clusters 2, 7 and 9 in Figure 4, Tables 2 and 4). However, the expression levels of classical UPR targets such as *IRE1*, *KAR2*, *HAC1*, *PDI1* and *EUG1* homologs were not altered in *A. gossypii* cells treated with 10 mM DTT, as confirmed by quantitative PCR (qPCR) (Additional file 3: Figure A3.1). Moreover, the *A. gossypii ERO1* and *LHS1* homologs, two other classical UPR targets, were transcriptionally down-regulated in *A. gossypii* (Additional file 2), as was the *GCN4* homolog, which has been previously shown to be induced by ER stress in other fungi [33, 36].

**Figure 5:**
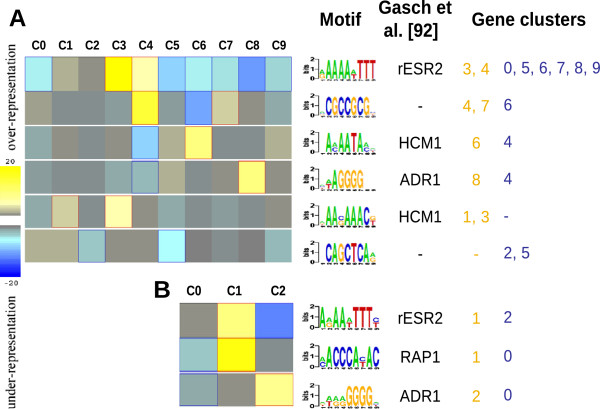
**Regulatory DNA elements significantly over- (yellow) and under-represented (blue) (p < 0.05) in different gene clusters.** In panel **A** are represented the co-expression clusters and in panel **B** the clusters of genes differentially up- (C1) and down-regulated (C2) by DTT. C0 comprises the genes that were not included in any of the other clusters.

A search for common regulatory DNA motifs in the promoter region of the DTT-regulated genes identified only 7 motifs that were common within one or more gene clusters (Figure 5), none of which matched known consensus binding sites for the transcriptional factors Hac1p or Gcn4p. Among the common promoter elements found, 4 were similar to known binding sequences for the *S. cerevisiae* transcription factors Rap1p, Adr1p and Hcm1p. The binding site for Rap1p, a positive transcriptional regulator for multiple growth related genes such as ribosomal protein genes [37], was over-represented in the DTT-induced gene cluster (Figure 5B). The consensus sequence for Adr1p binding was the only motif over-represented in the *A. gossypii* DTT down-regulated genes. Adr1p is a carbon source responsive zinc-finger transcription factor that is required for transcription of the glucose-repressed gene *ADH2*, of peroxisomal protein genes and of genes required for ethanol, glycerol, and fatty acid utilization [38]. Another had similarity to a Ribosomal RNA Processing Element (RRPE; AAAAATTT), to which the *S. cerevisiae* Stb3p has been demonstrated to bind [39] and that was identified by Gasch et al. [40] as being a common element found in the promoter region of several genes repressed during Environmental Stress Response (rESR). This motif was over-represented in the cluster of *A. gossypii* genes induced by DTT (Figure 5B) and also in the co-expression clusters 3 and 4 (Figure 5A), which are enriched in various functions associated with the RNA metabolism.

The expression of several genes involved in protein folding was significantly induced by DTT after 4 h of exposure, including *JEM1*, *SIL1*, *SSA2*, *STI1*, *SIS1*, *FES1*, *HSP104*, *HSC82*, *HSP82*, *AHA1*, *MDJ1*, *HSP78*, *APJ1* and *HSP26* homologs (Additional file 2). Some genes involved in the ERAD (*HRD1*, *USA1*, *UBX2* and *HLJ1* homologs) and proteasome degradation (*DOA4*, *UBP5*, *RPT3*, *UBA1*, *UBP2*, *CDC48*, *UFD1*, *DOA1* and *SHP1* homologs) were also significantly induced by DTT within 1 h of treatment. Up-regulation of vacuolar protein sorting (Cluster 9 in Figure 4, Tables 2, 4 and Additional file 2) and vesicle trafficking was observed in the DTT-treated cells (Table 4 and Additional file 2).

As mentioned above, DTT strongly and rapidly repressed the protein glycosylation pathway in *A. gossypii* (Table 3 and Additional file 2). The *A. gossypii YOS9* and *HTM1*/*MNL1* homologs, which in *S. cerevisiae* encode two proteins that are required for the ERAD of misfolded glycoproteins [41], were also repressed by DTT. Several genes involved in the COPI retrograde transport of proteins from the Golgi back to the ER were induced by DTT within 1 h of exposure (Additional file 2), which might have favoured the recycling of proteins. Some genes involved in the ER to Golgi protein trafficking were also induced by DTT, but COPII vesicle-mediated transport was strongly repressed by DTT (Additional file 2).

Genes involved in translation were down-regulated by DTT, but only after 4 h of treatment (Cluster 1 in Figure 4, Tables 2 and 3). DTT also repressed the transcription of the *A. gossypii SSH1* homolog, which in *S. cerevisiae* is involved in the co-translational translocation of proteins into the ER [42]. On the other hand, genes involved in mRNA degradation, such as *DOM34*, *KEM1*, *SKI2*, *SKI3*, *SKI7*, *LSM2* and *NMD2* homologs, were up-regulated by DTT within 1 h of treatment.

Thirty percent of the genes whose transcription was significantly decreased 30 min after DTT addition (p-value < 0.01 for a fold-change > 1.5) were predicted to encode secretory proteins (Additional file 1: Table A1.3). This decreased to 15% after 1 h and to 10% after 4 h of treatment. Less than 5% of the significantly up-regulated genes were predicted to encode secretory proteins.

## Discussion

The secretion of proteins by filamentous fungi is important for hyphal extension, degradation of substrates in natural ecosystems and pathogenicity [13, 43]. Thus, many filamentous fungi have evolved to secrete high amounts of proteins. Previous observations have suggested that the secretion abilities of *A. gossypii* were more similar to those of closely related yeast species than to those of other filamentous fungi [2]. Here, we observed that the total protein concentration in the supernatants of *A. gossypii* submerged cultures with sucrose as primary carbon source was indeed relatively low (≤ 218 mg/l). *In silico* analysis of the *A. gossypii* secretome predicted that it should represent around 1% of the total proteome, a percentage closer to that predicted for yeast secretomes (2-4%) [13, 44–47] than to that predicted for the secretomes of filamentous fungi (5-8%) [13, 48, 49]. However, in 2-D electrophoresis gel maps, 157–182 protein spots (corresponding to approximately 3-4% of the *A. gossypii* total proteome) could be detected in the supernatants of *A. gossypii* cultures, indicating that, although in low amount, this fungus secreted a variety of proteins through the plasma membrane, possibly more than computationally predicted.

The existence of different isoforms of the same proteins (e.g. different glycoforms) may have contributed to the higher number of protein spots observed in 2-D gels (up to 182) than that computationally predicted (54). Moreover, not all proteins require secretion signals to get out of a cell. Several fungal species have been reported to secrete large amounts of proteins that lack the typical secretion signals of conventionally secreted proteins, via alternative vesicular pathways (reviewed in [50]). Thus, some protein spots may correspond to proteins secreted via an alternative secretory pathway, which would fail to be predicted as secreted by the computational tools used. The possible contribution of intracellular proteins for some of the weakest spots detected can also not be ruled out. Nevertheless, the results from both experimental and computational analyses indicated that 1-4% of *A. gossypii* proteins are secreted.

Of the 54 proteins that were predicted to comprise the *A. gossypii* secretome, less than 33% were putative enzymes with hydrolytic activity. This is in line with the limited range of carbon sources which *A. gossypii* utilises [51, 52]. Extracellular lipase [53], amylase [54] and *β*-glucosidase [54] activities have previously been found in *A. gossypii* culture supernatants. In agreement with these observations, one putative lipase (AER454C) and two putative *β*-glucosidases (AGL354C and AGL343C) were predicted to be secreted by *A. gossypii*. However, neither of the putative *A. gossypii* amylases (AEL044W and AEL276C) were predicted to contain a N-terminal signal peptide and, thus, would not be expected to be secreted via the general secretory pathway.

Although extracellular protease activity in *A. gossypii* supernatants has been reported as negligible [2], nine putative proteases (Additional file 1: Table A1.2) were predicted to be secreted, the majority of which would probably be most active at acidic pH [55]. Given that the optimum pH range for *A. gossypii* is 6–7 [52] and that only low concentrations of proteins are secreted by this fungus, extracellular protease activity would indeed be expected to be low or undetectable. An invertase (AFR529W) was also predicted to be secreted by this fungus and subsequent experimental characterization of this protein confirmed its function and secretion into the culture supernatant [56]. Additionally, there was a putative acid phosphatase, a putative ureohydrolase and two putative FMN-binding proteins with probable oxidoreductase activity among the predicted secretome proteins, with no homologs in *S. cerevisiae*, but having homologs in *Kluyveromyces lactis*. Well-known extracellular proteins like *α*-factor mating pheromones (AAR163C and AFL062W) were also predicted to be secreted.

The fact that *A. gossypii* secretes a rather small number of proteins, together with the low concentration in which they are produced and the negligible extracellular protease activity could be advantageous in heterologous protein production if high secretion levels of recombinant proteins are achieved, since secreted products would be unlikely to be contaminated or degraded by the native proteins (*cf. Pichia pastoris* [47]). Moreover, a comprehensive analysis of the *A. gossypii* native secreted proteins could be used to facilitate product purification and quality control.

Despite these advantages, a major drawback of using *A. gossypii* for heterologous protein production is its low productivity [2]. Since *eglI* expression levels were much lower than those of the highly expressed *TEF* gene [57], we conclude that *eglI* was not highly overexpressed in the EGI producing cells and that a stronger promoter and/or better expression strategies would contribute to improving production and secretion of recombinant proteins by *A. gossypii*. Indeed, modifying the expression vector resulted in a 2 fold increase in extracellular EGI activity [54], while use of the *A. gossipii* native *TEF* promoter substantially improved production of recombinant *β*-galactosidase from *Aspergillus niger*, leading to relatively high levels of secretion [58]. Enhancement of the protein translation efficiency could also increase the production of secreted proteins, as indicated by the down-regulation of some genes involved in regulation of protein translation during EGI secretion.

Previous studies have shown that secretion of recombinant proteins can lead to secretion stress and trigger the UPR, which modulates both general and protein-specific transcriptional responses [6, 14, 36, 59]. This did not occur in *A. gossypii*, probably because *egl1* was not highly expressed and thus EGI production did not constitute a major burden to the cells. Carvalho et al. [60] recently shown that, in *A. niger*, the induction of the UPR pathway is dependent on the level of heterologous gene expression. Under relative low-expressing conditions, the basal protein folding and quality control machinery of the *A. niger* ER was adequate, but under high-expressing conditions ER stress was induced [60].

We induced secretion stress in *A. gossypii* cells with DTT, since it was not induced by EGI production. DTT has been widely used to induce secretion stress in investigations of the UPR [6, 30, 36, 59, 61–63], even though it is not a specific secretion stress inducer, inducing other stresses, such as oxidative stress, as well. Transcription of genes that may not be closely related to the UPR is also affected, as observed in a transcriptomic comparison of *Aspergillus nidulans* cells stressed by DTT treatment or by recombinant chymosin secretion, which showed similar changes in the expression of some genes, but not others [6]. However, DTT has consistently induced the UPR in various yeast and filamentous fungi [6, 30, 36, 59, 61–63].

DTT did not trigger a conventional UPR in *A. gossypii*, as the expression levels of several well-known UPR target genes (such as *IRE1*, *HAC1*, *KAR2*, *PDI1* and *EUG1*) remained unchanged and no UPRE-like motif was overrepresented in the gene clusters up-regulated by DTT. The amount of DTT used in this study (10 mM final concentration) was comparable to that used in several other studies with yeast and filamentous fungi (2–10 mM) [30, 61–63]. To our knowledge, only in studies of *A. nidulans* and *A. niger* has DTT been added to the cultures at a higher concentration (20 mM) [6, 59].

**Figure 6:**
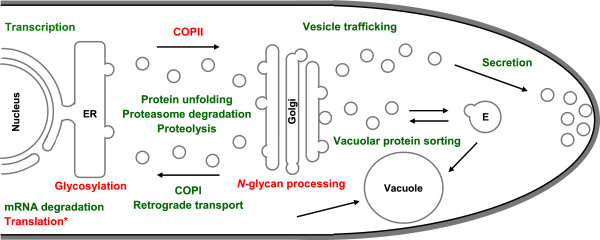
**Schematic representation of the** ***A. gossypii*** **protein secretory pathway with indication of relevant functions significantly up- (green) or down-regulated (red) by DTT-induced stress.** (*) Biological function down-regulated under recombinant EGI secretion conditions as well, (E) Endosome.

Although a classical UPR was not induced, there was evidence of secretion stress induced by DTT in *A. gossypii* cells (Figure 6). Expression of several genes involved in protein unfolding, ERAD, proteasome degradation, proteolysis, vesicle trafficking, vacuolar protein sorting and secretion significantly increased within 1 h of DTT treatment. In fungi, the UPR was thought to be exclusively dependent on Ire1p-mediated splicing of *HAC1* mRNA. However, an *IRE1*-, *HAC1*- and UPRE-independent pathway for transcriptional activation upon ER stress exists in *S. cerevisiae*, which may activate a core promoter through stimulation of RNA polymerase II holoenzyme activity [64]. Miyazaki et al. [28] have also demonstrated that *Candida glabrata* has lost the classic Ire1p-Hac1p UPR, but instead possesses an alternative mechanism, RIDD. In *A. gossypii*, the expression of several genes involved in mRNA degradation was induced by DTT, which suggests that a RIDD-like mechanism may exist in *A. gossypii* to reduce the ER load when there is secretion stress. Another mechanism to alleviate the load of proteins in the ER in some fungi is the transcriptional down-regulation of genes encoding secreted proteins in response to secretion stress (RESS). This down-regulation mechanism has been described in *T. reesei* [65], *A. niger* [66] and *S. cereviaise* [67]. In *A. gossypii*, DTT also repressed the transcription of a large number of genes encoding putative secretory proteins.

Two of the genes, encoding a subunit of the translocon complex (Ssh1p) and a chaperone involved in the translocation of newly synthesised proteins into the ER (Lhs1p), which were repressed by DTT may have contributed to accumulation of unfolded proteins in the cytosol. Intriguingly, most of the genes involved in protein folding that were up-regulated by DTT encoded cytosolic chaperones, co-chaperones and nucleotide exchange factors (Additional file 2). A stress response induced by misfolded cytosolic proteins that do not enter the secretory pathway, called UPR-Cyto, has been preliminarily characterized in *S. cerevisiae* [68, 69]. This cytosolic stress response induces the production of several cytosolic chaperones and co-chaperones. The UPR-Cyto response appears to be a specific *HSF1*-mediated module of the eukaryotic heat shock response [68, 69]. The transcript level of the *HSF1* homolog in DTT-stressed *A. gossypii* cells was only slightly increased. Moreover, no Hsf1p-like consensus binding sequence was overrepresented in the gene clusters analyzed. Thus, an UPR-Cyto may have been activated in *A. gossypii* in response to secretion stress induced by DTT, but probably not by Hsf1p.

Another striking difference in the *A. gossypii* transcriptional responses to DTT-induced stress, compared to that of *S. cerevisiae*, *A. niger* or *T. reesei*, was the rapid and severe down-regulation of the protein glycosylation pathway, an effect that at similar extent has only been described for treatments with tunicamycin. This could lead to an accumulation of improperly glycosylated proteins. In mammals, calnexin provides chaperone activity to retain incompletely glycosylated proteins in the ER, functioning as a component of the glycoprotein quality control system in the ER [70]. The *S. cerevisiae* homolog Cne1p also binds specifically to monoglucosylated oligosaccharides [71]. However, no homolog for the *CNE1* was found in the *A. gossypii* genome. An alternative mechanism of quality control should, therefore, exist to balance this absence.

Like the ER, the Golgi complex may also be involved in conformation-based disposal of abnormal proteins targeted for degradation [72]. Here we show that several post-ER pathways for protein disposal were up-regulated upon DTT treatment in *A. gossypii*. These included both the retrograde transport of proteins back to the ER for ERAD (via COPI-vesicle mediated transport) and protein transport via the endosomal system for degradation. COPII-vesicle mediated export of proteins from the ER to the Golgi was, however, down-regulated by DTT.

## Conclusion

Our results show that the lack of an active conventional UPR in *A. gossypii* may be compensated by alternative pathways, probably working simultaneously, to relieve the cells from secretion stress. The fact that *A. gossypii* has one of the smallest eukaryotic genomes known and, consequently, reduced genetic machinery, may have contributed to the differences between its transcriptional responses to secretion stress and those reported for other fungal species. The absence of a calnexin homolog in *A. gossypii* indicates that it lacks some of the ER quality control mechanisms of other fungi.

Despite the high genetic similarity it shares with *S. cerevisiae*, the regulation of the protein secretory pathway of *A. gossypii* and *S. cerevisiae* differed considerably. It must not be forgotten that significant sequence similarity between *A. gossypii* and *S. cerevisiae* is restricted to coding regions [73] and that regulatory genes are reported to show a higher evolutionary rate than structural genes, resulting in homologous transcriptional factors playing different regulatory functions in different organisms [74, 75]. Therefore, although earlier results have shown that the *A. gossypii* protein secretion potential is more similar to yeast than to other filamentous fungi, reflecting phylogentic relationships rather than morphology, the differences in regulation may suggest novel ways of improving protein secretion in *A. gossypii*.

## Methods

### Strains and culture conditions

*A. gossypii* ATCC 10895, kindly provided by Prof. P. Philippsen (Biozentrum, University of Basel, Switzerland) and here referred to as the parental strain, was used for proteomic analyses. A recombinant *A. gossypii* EGI producing strain (VTT D-101398) and its corresponding empty vector control strain described in Ribeiro et al. [2] were used for transcriptomic analyses. Stock cultures were maintained as spores suspended in 20% (v/v) glycerol, 0.8% (w/v) NaCl with 0.025% (v/v) Tween 20 at −80°C.

Pre-cultures to inoculate bioreactors were grown in 250 ml Erlenmeyer flasks containing 50 ml of AFM (1% (w/v) yeast extract, 1% (w/v) tryptone, 2% (w/v) glucose and 0.1% (w/v) myo-inositol), which was supplemented with 200 μg/ml G418 (Sigma) for maintenance of the recombinant strains. *A. gossyppii* pre-cultures were inoculated with 10^6^ spores and grown for 14–17 h at 30°C and 200 rpm.

For cultivation of the recombinant strains, Biostat® CT bioreactors, maximum working volume of 2.5 l (B. Braun Biotech International, Sartorius AG), containing 1.5 l or 2 l of AFM plus 200 μg/ml G418 were used. Biostat® B-DCU bioreactors, maximum working volume of 2 l (B. Braun Biotech International, Sartorius AG), were used for cultivation of the parental strain in 1 l of either modified AFM or defined minimal medium [76], both containing 2% (w/v) sucrose as carbon source instead of glucose. Bioreactors were inoculated to an initial biomass of 0.13 ± 0.08 g/l, for recombinant strains, or 0.60 ± 0.05 g/l, for the parental strain. Cultures were grown at 30°C and 500 rpm, with 1.0 volume of gas per volume of culture per minute (vvm) aeration. Culture pH was kept at 6.0 ± 0.1 by the addition of 1 M KOH or 1 M H_3_PO_4_. Polypropylene glycol (mixed molecular weights) [77] was added to prevent foaming. Gas concentration (CO_2_, O_2_, N_2_ and Ar) was analyzed continuously in an Omnistar quadrupole mass spectrometer (Balzers AG), calibrated with 3% CO_2_ in Ar.

For dry weight determination, culture samples were filtered through pre-dried and pre-weighed Whatman GF/B glass fibre filters, washed with at least two sample volumes of double-distilled water and dried to a constant weight at 105°C. Aliquots of the culture filtrates were stored at −20°C.

Residual sugars and produced metabolites in the culture filtrates were quantified by high performance liquid chromatography (HPLC) as previously described [78]. Total protein concentration in the cell-free broth was measured using the Thermo Scientific Pierce Coomassie (Bradford) Protein Assay kit, with bovine serum albumin (BSA) as standard. The activity of secreted EGI in the culture filtrates was determined as described in Ribeiro et al. [2], using 4-methylumbelliferyl-β-D-lactoside (MULac) (Sigma) as substrate. Volumetric EGI enzyme activity was defined as micromoles of 4-methylumbelliferone (MU) (Sigma) formed per minute and per litre of culture (μmol min^−1^ l^−1^) under the assay conditions.

For gene expression analysis, mycelial samples from the recombinant strains were collected from duplicate bioreactor cultivations 4 h, 7 h, 10 h and 13.5 h after inoculation. After 9.5 h, DTT was added to two out of four *A. gossypii* VTT D-101398 cultures at a final concentration of 10 mM. Samples from DTT-treated cultures were collected 30 min, 1 h and 4 h after DTT addition. Mycelium was rapidly separated from the culture supernatant by filtration through Whatman GF/B glass fibre filters, washed with two sample volumes of 0.9% (w/v) NaCl, frozen immediately in liquid nitrogen and stored at −80°C.

### *In silico*secretome prediction

A computational approach similar to those described to predict the secretomes of *Candida albicans* [44], *K. lactis* [45, 46], *P. pastoris* [47] and *Trichoderma* species [49] was used to analyze the putative protein sequences of the 4,776 ORFs annotated in the *A. gossypii* genome (ftp://ftp.ncbi.nlm.nih.gov/genomes/Fungi/Eremothecium_gossypii_uid10623/, accessed on December 2012). In this protocol, SignalP version 3 [79] was used to identify the presence of N-terminal signal peptides and TMHMM version 2 [80] was used to identify putative transmembrane regions in proteins with putative signal peptides. Only proteins with signal peptides predicted by the SignalP Neural Networks and Hidden Markov Models were included. Proteins with 1 predicted transmembrane spanning region were kept in the dataset if it was located in the N-terminal region before the predicted signal peptide cleavage site. Sequences with more than 1 transmembrane spanning region were excluded. TargetP version 1.1 [10] and the fungal version of big-PI [81] were then used to eliminate proteins predicted to be targeted to the mitochondrion and/or to contain a GPI anchor. Finally, WoLF PSORT [11] was used for sub-cellular localization prediction. The default value for the total number or nearest neighbors (*k*) was 27 and only proteins with a *k* > 13 for extracellular location were included in the secretome. The NetNGlyc version 1.0 server [http://www.cbs.dtu.dk/services/NetNGlyc/] was used to predict *N*-glycosylation sites and the databases UniProt (http://www.uniprot.org) [82], CAZy (http://www.cazy.org) [83] and *MEROPS* (http://merops.sanger.ac.uk) [55] were used to retrieve predicted functions for *A. gossypii* putative proteins. The EMBOSS pepstats application (http://emboss.bioinformatics.nl/cgi-bin/emboss/pepstats) [84] was used to calculate the theoretical molecular weight and isoelectric point for each putative protein.

### Gel electrophoresis of secreted proteins

The proteins present in 15 μl of culture supernatant were analyzed by 12% (w/v) SDS-PAGE followed by silver staining.

For 2-D gel electrophoresis, the total proteins in parental strain culture filtrates collected at the beginning of the stationary phase were precipitated overnight at −20°C in 10% (w/v) trichloroacetic acid and 66.6% (v/v) acetone. The pellet was washed with ice-cold acetone, dried and resuspended (15 min at room temperature) in 2-D sample solution (8 M urea, 10 mM DTT, 2% (v/v) Pharmalyte 3–10 (GE Healthcare) and 2% (v/v) Triton X-100). Insoluble material was removed by centrifugation and the protein concentration in the samples was determined using the 2-D Quant Kit (GE Healthcare). Each sample was independently prepared and used for duplicate 2-D electrophoresis analyses.

Equal amounts of total extracellular protein (100 μg) were cup-loaded in Immobiline DryStrip gel strips pH 3–10, 18 cm (GE Healthcare) previously rehydrated and subjected to isoelectric focusing (IEF) using an Ettan IPGphor II (GE Healthcare) according to the instructions of the manufacturer. The first-dimension isoelectric focusing was followed by second-dimension 11% (w/v) SDS-PAGE using an Ettan DALT electrophoresis system (GE Healthcare). After electrophoresis, the gels were fixed for 30 min with 30% (v/v) ethanol and 0.5% (v/v) acetic acid in water and subsequently stained with SYPRO Ruby (Bio-Rad) according to the instructions of the manufacturer. The 2-D gels were scanned in a Typhoon 8610 variable mode imager (GE Healthcare) at 300 dpi resolution and gel images analysed with Melanie software version 7.0 (Geneva Bioinformatics (GeneBio) SA). After automatic spot detection, artefacts were manually removed and the weaker spots (< 0.1% of the whole gel volume) were eliminated. The remaining spots were then linked to allow comparison between samples.

### RNA extraction and gene expression analysis

Total RNA extraction from frozen mycelium was carried out using the RNeasy Plant Mini kit (QIAGEN) according to the manufacturer’s instructions for isolation of total RNA from filamentous fungi. RNA concentration and purity were determined using a NanoDrop ND-1000 (NanoDrop Technologies) and integrity of RNA was analyzed using an Agilent 2100 Bioanalyzer (Agilent Technologies).

For microarray analysis, custom-made *A. gossypii* gene expression 12×135K arrays were designed and manufactured by Roche NimbleGen. Each slide contained 12 independent arrays, each comprising four replicates of 33,364 probes covering 4,758 ORFs of *A. gossypii* and the *T. reesei egl1* gene (7 probes/target ORF). 10 μg of total RNA were used for reverse transcription and synthesis of cDNA using the SuperScript II Double-Stranded cDNA Synthesis Kit (Invitrogen) according to the Roche NimbleGen Arrays User’s Guide: Gene Expression Arrays v5.0, available from the NimbleGen website [http://www.nimblegen.com]. The cDNA was quantified in a NanoDrop ND-1000 (NanoDrop Technologies) and its integrity analyzed using an Agilent 2100 Bioanalyzer (Agilent Technologies). The double-stranded cDNA was labelled with Cy3 fluorescent dye, hybridized to the custom-made microarray slides (Roche NimbleGen) and scanned using a NimbleGen MS 200 Microarray Scanner (Roche NimbleGen) according to the instructions of the manufacturer.

For qPCR, 1 μg of total RNA was reverse transcribed using the NZY First-Strand cDNA Synthesis Kit (NZYTech) and qPCR analyses were performed as previously described [56, 85], with the primer pairs shown in Additional file 3: Table A3.1.

### Microarray data analysis

The raw array data obtained from NimbleScan software version 2.5.26 (Roche NimbleGen) was preprocessed with the Robust Multichip Average (RMA) method [86]. Array data quality was controlled with arrayQualityMetrics [87] and sample wise Principal Component Analysis (PCA) of raw, RMA preprocessed and repeat averaged data. Linear Models for Microarrays (LIMMA) [88] was subsequently used to select significantly changing genes with a cut-off of p-value < 0.01 (which corresponds to a false discovery rate (FDR) of 1% in this analysis) and fold-change > 1.5. For each gene its Pearson correlation with EGI activity (μmol min^−1^ l^−1^) was calculated. The FDR of these correlations (4.4% for absolute correlation > 0.7) was estimated from the Q-value [89] using the R package ‘qvalue’.

Each gene’s repeat averaged expression values over all the samples i.e. expression profiles were clustered with R-package ‘Mfuzz’ [90] with parameter m set to 1.35 and number of clusters to 9. Parameters were selected based on visual evaluation of cluster profiles. Genes with cluster membership > 0.7 were assigned to a co-expression cluster for further analysis.

*A. gossypii* gene mappings to *S. cerevisiae* genes from the *Ashbya* Genome Database (http://agd.vital-it.ch) [91] were used to map the array results of differential gene expression, gene expression correlation with EGI activity (μmol min^−1^ l^−1^) and co-expression clusters to *S. cerevisiae*. GO class analyses used *S. cerevisiae* GO annotations. The R-package ‘GSA’ [92] was used for the GO analysis of differential expression of genes, the R-package ‘GOstats’ [93] was used to analyse gene expression correlation and co-expression clusters and REVIGO [94] was used to summarize the GO term lists.

Promoter sequences were analysed with the tool Finding Informative Regulatory Elements (FIRE) [95], with default settings using the *A. gossypii* genome. Co-expression clusters and clusters of differentially expressed genes were used as groups of genes from which to find common promoter elements. Genes which were not assigned to any cluster were assigned to group number 0 for this analysis. As promoter we used 1500 bases upstream from the putative ORF of each gene. The identified promoter elements were mapped with FIRE to the known promoter elements described in Gasch et al. [96] in order to annotate them.

### Availability of supporting data

The data sets supporting the results of this article are included within the article (and its additional files). The raw microarray data was deposited in GEO with accession number GSE62366.

## Electronic supplementary material

Additional file 1: ***A. gossypii*** **secretome and microarray gene expression analysis data. Table A1.1.** *Secretome analysis data.* According to our protocol proteins were predicted to be secreted if: (a) contained a signal peptide predicted by both SignalP methods (NN D-score SP=“Y” and HMM Pred=”S”); (b) did not contain predicted transmembrane spanning regions (PredHel=”0”) or this was located in the N-terminal region before the predicted signal peptide cleavage site (PredHel=“1*”); (c) were predicted not to be targeted to the mitochondrion (loc≠”M”); (d) did not contain predicated GPI anchors (big-PI Quality=”P” or “P or S”); and (e) presented a k>13 for extracellular location predicted by WoLF PSORT. **Table A1.2.** *A. gossypii predicted secretome. A. gossypii* ORFs encoding the putative proteins predicted to be secreted to the extracellular space and corresponding *S. cerevisiae* homologs. Predicted functions, number of *N*-glycosylation sites, theoretical molecular weights (MW) and isoelectric points (pI) are also indicated. **Table A1.3.** *Microarray gene expression analysis data.* “Best Cluster” show the co-expression cluster to which genes were assigned (cluster membership>7) after clustering their expression profiles with the R-package ‘Mfuzz’ (m=1.35, 9 clusters). “log2(fold change)” show the fold change of the averaged RMA preprocessed signal of each gene’s repeat upon DTT treatment or in EGI producing vs. non-EGI producing cultures at the indicated times. “Significance” show the results of a significance test (R package LIMMA, p-value<0.01, log2 fold change>0.58), 1 indicates up-regulation and −1 down-regulation. “q-value”, “p-value” and “Absolute correlation > 0.7” show the results from the Pearson correlation of each gene’s expression to EGI activity (μmol min^-1^ l^-1^) in EGI producing conditions, 1 indicates up-regulation and −1 down-regulation during EGI production (FDR of 4.4% for absolute correlation>0.7). “NN D-score SP” and “HMM Pred” indicate putatively encoded secretory proteins as predicted by both SignalP methods (NN D-score SP=”Y” and HMM Pred=”S/A”). (XLS 4 MB)

Additional file 2: **Secretion-related** ***A. gossypii*** **genes with significant fold changes (** ^**a**^ **p-value < 0.01) in their transcript levels after treatment with DTT for 30 min, 1 h and 4 h.** The corresponding *S. cerevisiae* homologs are indicated, as well as predicted functions. Green indicates up-regulation and red down-regulation by DTT. (XLS 53 KB)

Additional file 3: **qPCR results of selected genes and primer sequences used. Figure A3.1.** *Expression analysis of selected genes by qPCR in the EGI expressing strain immediately before (DTT 0 h) and 1 h after addition of DTT (DTT 1 h).* No significant changes (p > 0.2) were observed in the expression of the selected genes 1 h after addition of DTT. The gene expression levels were normalized to the expression level of *AgACT1*. Data represents the mean ± standard deviation of two independent bioreactor cultures. Each cDNA sample was analyzed in triplicate and the coefficient of variation between the results for these technical replicas was < 30%. **Table A3.1.** *Primers used in the qPCR analysis.* (PDF 86 KB)

## Abbreviations

2-D: Two-dimensional
AFM: *Ashbya* full medium
bZIP: Basic-leucine zipper
DMM: Defined minimal medium
DTT: Dithiothreitol
ER: Endoplasmatic reticulum
EGI: Endoglucanase I
ERAD: ER-associated degradation
FDR: False discovery rate
FIRE: Finding informative regulatory elements
GO: Gene ontology
LIMMA: Linear models for microarrays
qPCR: Quantitative PCR
RMA: Robust multi-array average
RIDD: Regulated *IRE1*-dependent decay
SDS-PAGE: Sodium dodecyl sulfate polyacrylamide gel electrophoresis
UPR: Unfolded protein response
UPRE: UPR elements
Vvm: Volume of gas per volume of culture per minute.
