# Supplementary material for: Investigation of protein secretion and secretion stress in Ashbya gossypii
Source: BMC Genomics. 2014 Dec 18;15(1):1137. doi: 10.1186/1471-2164-15-1137 (PMC4320514; doi:10.1186/1471-2164-15-1137)
Supplement: Supplementary file 3 — Additional file 3: qPCR results of selected genes and primer sequences used. Figure A3.1. Expression analysis of selected genes by qPCR in the EGI expressing strain immediately before (DTT 0 h) and 1 h after addition of DTT (DTT 1 h). No significant changes (p > 0.2) were observed in the expression of the selected genes 1 h after addition of DTT. The gene expression levels were normalized to the expression level of AgACT1. Data represents the mean ± standard deviation of two independent bioreactor cultures. Each cDNA sample was analyzed in triplicate and the coefficient of variation between the results for these technical replicas was < 30%. Table A3.1. Primers used in the qPCR analysis. (PDF 86 KB) [file 12864_2014_6951_MOESM3_ESM.pdf]

Additional File 3: qPCR results of selected genes and primer sequences used.

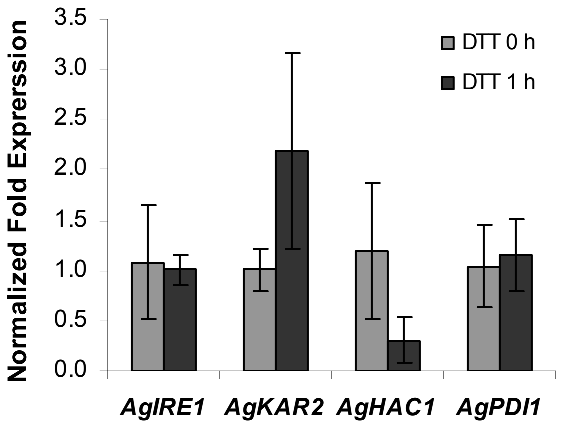

Figure A3.1 *Expression analysis of selected genes by qPCR in the EGI expressing strain immediately before (DTT 0 h) and 1 h after addition of DTT (DTT 1 h).* No significant changes ($p > 0.2$) were observed in the expression of the selected genes 1 h after addition of DTT. The gene expression levels were normalized to the expression level of *AgACT1*. Data represents the mean ± standard deviation of two independent bioreactor cultures. Each cDNA sample was analyzed in triplicate and the coefficient of variation between the results for these technical replicas was < 30%.

Table A3.1 *Primers used in the qPCR analysis.*

**Gene**	**Primers**	
	**Forward**	**Reverse**
AgIRE1	ATGGCGAAGTGGGTTTCTCCAA	TTCCGGTGGTTTCAAGGGATGA
AgKAR2	AAGAAGGCGTCCAAGGGCATTA	AACACCTTCCTCGCCAGACAAA
AgHAC1	CCAAGACGCAGGAGGAGAAGGA	ACTTGCGCTCCAGGTACAGCA
AgPDI1/EUG1	ACCACCCACGACCAAATTGTCA	ATCCGCAAGCTCCTCAAAGGTT
AgACT1	TCGTTGCCACACGCCATTT	AAAGGAGTAGCCACGTTCCGATAG
